# Diseases of the Nervous System

**Published:** 1906-03-31

**Authors:** 


### PROGRESS IN MEDICINE AND SURGERY.

### DISEASES OF THE NERVOUS SYSTEM.

(*Continued from page* 423.)

**Meningitis.**—During recent years the study of meningitis has made considerable advances, especially since the introduction of lumbar puncture has shown the presence of a variety of micro-organisms as exciting factors. Interest has also been stimulated by the occurrence lately of numerous small epidemics of the form known as cerebro-spinal meningitis. The comparative frequency and diagnostic value of certain symptoms has been studied by Howard Tooth,[10] his observations being based on a record of 65 cases occurring in the wards of St. Bartholomew's Hospital. The pathological aspects of the same cases are discussed by T. Horder.[11] Only four of these cases were over twenty years of age, and, except in the epidemic form, the disease is especially one of early childhood. The chief varieties met with are the tuberculous, the meningococcal, the pneumococcal, and the streptococcal infections. Cases in which the meningococcus are found include those classed as posterior basic, simple meningitis of children, sporadic cerebro-spinal, and epidemic cerebro-spinal meningitis. Whether the same identical diplococcus meningitidis is present in all these forms has not yet been determined. The tuberculous form appears to be practically always secondary to some tuberculous focus elsewhere, often in children a caseating bronchial or mesenteric gland, more rarely one of the cervical group. Tubercle bacillus is the commonest cause, being found in almost 50 per cent. of cases of acute meningitis. The mode of entrance of the infective organism in other cases is not always clear. Occasionally pneumonia or a general septic infection present, and otitis media is a not at all infrequent forerunner of the onset of meningitis. Attention is also becoming more and more directed to the state of the nose and its accessory sinuses and cavities as furnishing the clue to infection. The importance in this connection of a more careful and systematic study of all nasal and bronchial secretions is emphasised by C. N. B. Camac, of Cornell University, and by St. Clair Thomson.[12] The latter points out that micro-organisms in the depths of the nose are always pathological, as, in health, although the vibrissæ and vestibules of the nose are swarming with micro-organisms of many sorts, these rapidly decrease as the nasal chambers are entered, so that the depths of the nose and nasopharynx are quite sterile. In autopsies in intracranial diseases, systematic examination of these parts is often omitted, perhaps from a needless fear of disfiguring the cadaver. Of ocular symptoms, optic neuritis is common in tuberculous cases, but much less frequent in those belonging to the meningococcus group in which, however, a temporary amaurosis is comparatively common. Retraction of the upper eyelid, similar to that observed in Graves' disease, is a common symptom which is not so often mentioned, however, in textbooks. The cranial nerves and their nuclei escape the inflammation surrounding them in a rather surprising manner, with the exception of those of the ocular muscles which are apt to be involved, as shown by the frequency of squints. The sixth nerve is particularly liable to involvment, hence internal strabismus is the commonest form. Squints are naturally commonest in meningitis of the base, hence they are observed oftener in the meningococcus than in the tubercular form. Other paralytic symptoms are rare, but muscular spasm or hypertonus is a prominent feature in affections of the meninges. One of its earliest manifestations is slight stiffness of the muscles of the neck, developing later into cervical, or, in extreme cases, general opisthotonus. A similar affection of the abdominal muscles produces the scaphoid abdomen. Tonic spasm of the limbs or trunk appears less common in the pneumococcal cases—owing perhaps to the shorter course these usually run. It is very frequent in tubercular infections, and especially prominent in those due to the meningococcus. Extreme persistent opisthotonos rarely occurs except in the last named. This phenomenon of meningeal rigidity is complex, but " must be due to some disturbance of balance between controlling mechanisms in the cerebellum, optic thalamus, and corticopyramidal systems, which in health maintain the muscular tone." Passive flexion or extension may be resisted and be painful. Where this affects the hamstring muscles, passive extension of the knee being resisted and causing severe pain if the thigh is flexed to a right angle with the trunk, it constitutes the sign of Kernig, who first directed attention to it in 1884. But little notice was taken of his description, however, until in 1898 Netter examined for and found it in an epidemic of cerebro-spinal meningitis in Paris. Various explanations of the phenomenon have been given. Hassin,[13] combining several of these, suggests that it is due to irritation of the spinal roots, produced by the stretching of the sciatic nerve. It is not absolutely pathognomonic of meningitis as it may be elicited also in sciatica, typhoid fever, and certain cerebral conditions such as cerebellar tumour, sinus thrombosis, and hæmorrhage. It is, moreover, occasionally absent in tubercular and other forms of meningitis. But on the whole it is a very constant and sure sign, and the absence of meningeal irritation cannot be proved in all the cases apart from meningitis in which it does occur. The reflexes, North finds, are very variable. Not infrequently they are absent altogether, even in cases of intense hypertonia, and in four of his cases absent knee-jerks were observed with an extensor plantar response—an apparent contradiction. Fever is a very variable symptom. It commonly has an irregular oscillatory character. Wide daily oscillations and persistent rise are especially marked in the posterior basic and other meningococcus forms in which a pseudo-hectic type is often produced.

S. West [14] has drawn attention to the peculiar type of respiration which occurs in the disease, of which he has noticed two periodic forms, namely, the ordinary Cheyne-Stokes type, characterised by long pauses, crescendo and diminuendo of respiration, and extraordinary rapidity of breathing, and a grouped form of periodic breathing in which groups of two or three extremely slow respirations are separated by pauses of ten or fifteen seconds. Cheyne-Stokes breathing, S. West thinks, is always fatal, but grouped respiration has occurred in cases which recovered. Meningitis due to the pneumococcus runs a virulent and rapid course. The average duration of tubercular cases is longer, about fourteen days, but these, too, always end in death, and recovery only seems to occur in some cases of infection with the meningococcus. Horder thinks that all cases tend to involve the spinal as well as the cerebral meninges, so that meningitis is really always cerebro-spinal, but the particular variety usually distinguished by this name has certain characteristics of its own and in seasonal incidence, infectivity and contagiousness, and occasional epidemic outbreaks closely resembles pneumonia. This resemblance is increased, as Osler [15] points out, by much similarity between the pneumococcus and the meningococcus. Clinically the general features of meningitis are present with special prominence of those due to spinal implication such as cervical opisthotonos. Purpura, herpes and other skin lesions have occurred so widely in some epidemics as to give rise to the name of spotted fever. Of Osler's cases 17 out of 32 recovered. Montague Murray draws attention to the variability of the signs and the lengthy nature of cases which may yet be followed by recovery, in one case after unconsciousness had persisted for ten months. T. F. S. Caverhill states that during epidemics patients may go to bed well and be found dead in the morning. In a recent epidemic in a Northamptonshire village [16] household infection was rather clearly shown, in one case seven out of twelve, and in another five out of eight persons in the same house being attacked, but the periods of infection, invasion, and incubation were so extremely irregular that the outbreak furnishes no definite evidence of infection from person to person, although personal infection was highly probable and may have been effected through the channel of nasal mucus.[17]

[10] Brit. Med. Jour., Oct. 21. [11] Ibid. [12] Ibid., pp. 1016 and 1017. [13] Med. Record, Sept. 9. [14] Brit. Med. Jour., Oct. 21, p. 1017. [15] Ibid. [16] Lancet, Sept. 30, p. 973. [17] Brit. Med. Jour., Oct. 7, p. 895.

